# Comprehensive Target Screening and Cellular Profiling of the Cancer-Active Compound b-AP15 Indicate Abrogation of Protein Homeostasis and Organelle Dysfunction as the Primary Mechanism of Action

**DOI:** 10.3389/fonc.2022.852980

**Published:** 2022-04-22

**Authors:** Johannes Gubat, Karthik Selvaraju, Linda Sjöstrand, Dhananjay Kumar Singh, Maria V. Turkina, Bernhard Schmierer, Pierre Sabatier, Roman A. Zubarev, Stig Linder, Pádraig D’Arcy

**Affiliations:** ^1^ Department of Biomedical and Clinical Sciences, Linköping University, Linköping, Sweden; ^2^ Department of Pharmacy, Central University of South Bihar, Gaya, India; ^3^ Department of Medical Biochemistry and Biophysics, Division of Chemical Biology, Karolinska Institutet, Stockholm, Sweden; ^4^ Department of Medical Biochemistry and Biophysics, Division of Physiological Chemistry I, Karolinska Institutet, Stockholm, Sweden; ^5^ Department of Pharmacological and Technological Chemistry, I.M. Sechenov First Moscow State Medical University, Moscow, Russia; ^6^ Department of Oncology-Pathology, Karolinska Institutet, Stockholm, Sweden

**Keywords:** b-AP15, proteasome inhibitor, mitochondrial dysfunction, dienone, Michael acceptor, target screening

## Abstract

Dienone compounds have been demonstrated to display tumor-selective anti-cancer activity independently of the mutational status of TP53. Previous studies have shown that cell death elicited by this class of compounds is associated with inhibition of the ubiquitin-proteasome system (UPS). Here we extend previous findings by showing that the dienone compound b-AP15 inhibits proteasomal degradation of long-lived proteins. We show that exposure to b-AP15 results in increased association of the chaperones VCP/p97/Cdc48 and BAG6 with proteasomes. Comparisons between the gene expression profile generated by b-AP15 to those elicited by siRNA showed that knock-down of the proteasome-associated deubiquitinase (DUB) USP14 is the closest related to drug response. USP14 is a validated target for b-AP15 and we show that b-AP15 binds covalently to two cysteines, Cys203 and Cys257, in the ubiquitin-binding pocket of the enzyme. Consistent with this, deletion of USP14 resulted in decreased sensitivity to b-AP15. Targeting of USP14 was, however, found to not fully account for the observed proteasome inhibition. In search for additional targets, we utilized genome-wide CRISPR/Cas9 library screening and Proteome Integral Solubility Alteration (PISA) to identify mechanistically essential genes and b-AP15 interacting proteins respectively. Deletion of genes encoding mitochondrial proteins decreased the sensitivity to b-AP15, suggesting that mitochondrial dysfunction is coupled to cell death induced by b-AP15. Enzymes known to be involved in Phase II detoxification such as aldo-ketoreductases and glutathione-S-transferases were identified as b-AP15-targets using PISA. The finding that different exploratory approaches yielded different results may be explained in terms of a “target” not necessarily connected to the “mechanism of action” thus highlighting the importance of a holistic approach in the identification of drug targets. We conclude that b-AP15, and likely also other dienone compounds of the same class, affect protein degradation and proteasome function at more than one level.

## Introduction

**Figure 1:**
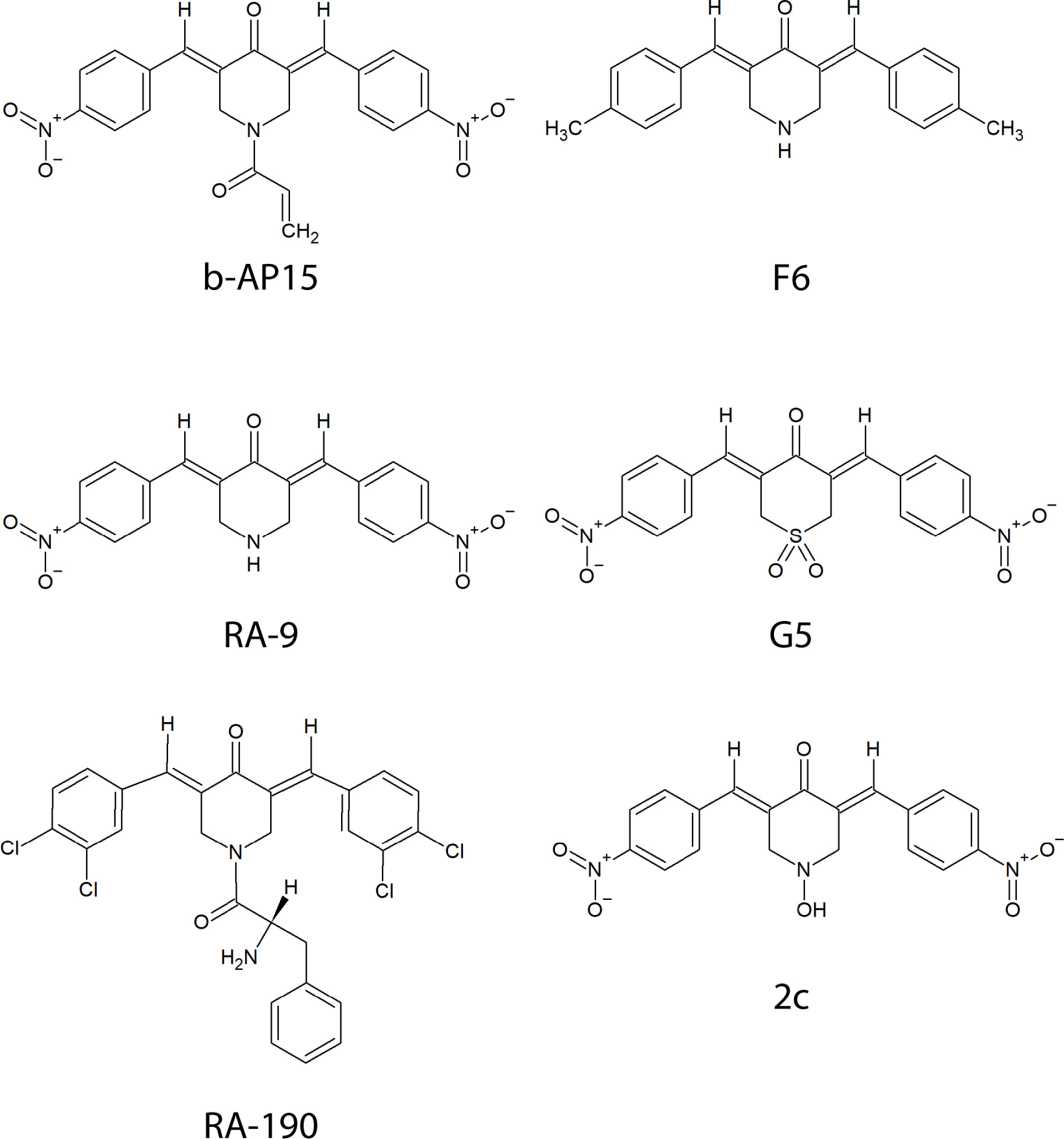
Chemical structures of compounds containing a bioactive 1,5-diaryl-3-oxo-1,4-pentadienyl pharmacophore described in the text.

Altered protein homeostasis is a common feature of many malignancies. Genetic alterations and the demands of incessant growth signaling lead to increased protein synthesis and production of misfolded proteins. Approximately 80% of protein degradation is regulated by the ubiquitin-proteasome system (UPS), and as such has emerged as an important target in malignancies (1, 2). In the previous decade, proteasome inhibitors have been successfully employed in the clinic, and agents that inhibit other UPS components are in various stages of development (2).

b-AP15 was discovered in a screen for agents that induce cathepsin-dependent apoptosis in cancer cells (3). Like other compounds (**Figure 1**) with a 1,5-diaryl-3-oxo-1,4-pentadienyl pharmacophore, it inhibits ubiquitin-isopeptidase activity, causes accumulation of polyubiquitinated protein conjugates, and induces p53-independent cell death (3, 4). The initial studies on b-AP15 had encouraging results. Potent anti-cancer activity was observed using several cultured tumor cell lines and different mouse tumor models (5). The relative insensitivity of peripheral blood mononuclear cells and immortalized epithelial cells compared with cancer cell lines suggested selectivity for cancer and a workable therapeutic window (5). Multiple studies have since shown activity in various models for hematologic and solid tumors notably including Ewing’s sarcoma, bortezomib-resistant multiple myeloma, and mantle cell lymphoma which are associated with poor prognosis (5–14).

Two pieces of evidence point to UPS inhibition as a mechanism of action for b-AP15. Firstly, experiments in a cell line expressing Ub^G76V^-YFP as a fluorescent reporter for ubiquitin accumulation show development of fluorescence followed abruptly by cell death in response to b-AP15 treatment in a manner similar to that observed with the proteasome inhibitor bortezomib (5, 15, 16). Secondly, the transcriptomic response of cells treated with b-AP15 is similar to previously described proteasome inhibitors, such as MG-262 and bortezomib (5, 17, 18). At the proteome level, b-AP15 and bortezomib also show a similar signature (18). One notable difference was that b-AP15 induced a more potent chaperone response, possibly in connection with the accumulation of high molecular weight polyubiquitin accumulation, compared to bortezomib (5). Although the mechanism for the observed UPS inhibition has not been completely elucidated, we found that b-AP15 selectively inhibited the proteasome-associated deubiquitinases (DUBs) USP14 and UCHL5 at IC_50_-IC_90_ concentrations. The accumulation of polyubiquitin on simultaneous siRNA-mediated knockdown of USP14 and UCHL5 has been observed earlier by DeMartino and colleagues (19), which we subsequently confirmed (20).

The presence of an α,β-unsaturated dienone in b-AP15 (**Figure 1**) suggests that the compound could react with multiple targets. α,β-unsaturated carbonyls are known to alkylate cysteines *via* Michael addition *in vitro* and in complex proteomes (21, 22). Several alternative/complementary mechanisms of action have been proposed for compounds having the 1,5-diaryl-3-oxo-1,4-pentadienyl pharmacophore such as induction of oxidative stress through mitochondrial dysfunction (23) and interference with Wnt signaling (10). A recent study on VLX1570, a b-AP15 analog, that employed activity-based proteomic profiling showed several off-target effects including aggregation of CIAPIN1/anamorsin, a component of the electron transport chain that exerts anti-apoptotic effects (24). A study on RA190, a compound with the same pharmacophore as b-AP15, also showed interaction with multiple proteins (25).

In this study, we explore mechanisms of action and targets of b-AP15 using different approaches. Our findings are consistent with previous results implying proteasome inhibition and proteotoxic stress as the main mechanisms of action and the proteasome-associated deubiquitinase USP14 as a target of b-AP15. Using proteomics and CRISPR/Cas9 loss-of-function screening we define other b-AP15 interacting proteins and extend previous findings with regard to the mechanism of cytotoxicity of the compound.

## Materials and Methods

### Cell Culture and Treatments

The colon carcinoma HCT116 cell line was cultured in McCoy’s 5A (modified) medium supplemented with GlutaMAX™ (ThermoFisher), 10% fetal bovine serum (Gibco), and penicillin-streptomycin (Gibco). Breast cancer cell line MCF7 used for the gene expression analysis was cultured in Minimum Essential Eagle medium with 1 mM sodium pyruvate medium supplemented with GlutaMAX™ (ThermoFisher), 10% fetal bovine serum, and penicillin-streptomycin. HEK293 Rpn11-HTBH cells were cultured in Dulbecco’s modified Eagle’s medium (DMEM) supplemented with GlutaMAX™ (ThermoFisher), 10% fetal bovine serum, and penicillin-streptomycin. B16-F10 mouse melanoma cells (ATCC) were maintained in DMEM (Lonza) supplemented with 10% FBS (Gibco) and 2 mM L-glutamine (Lonza). The cells were maintained at 37°C in a 5% CO_2_ humidified incubator and were regularly passaged at 70-80% confluence. Treatments with b-AP15(Vivolux AB), bortezomib (Sigma), and chloroquine (Sigma) were all carried out at 70-80% confluence. All the compounds used were dissolved in DMSO. Treatment and control groups were exposed to an equivalent amount of DMSO (0.1-0.5%).

### Degradation of Long-Lived Proteins

The degradation rate of long-lived proteins was measured as previously described (26). Briefly, HCT116 cells were grown in 6-well plates and labeled by incubating with complete medium containing ^3^H-phenylalanine (PerkinElmer) at a concentration of 5µCi/ml for 20 hours at 37°C in a humidified incubator. The radioactive medium was then removed then the cells were washed twice with chase medium (complete medium added with 2mM phenylalanine) and incubated in chase medium containing 1 µM b-AP15, 1 µM bortezomib, or 40 µM chloroquine singly or in combination for 2 hours. The medium was then removed and replaced with 2mL fresh chase medium containing the respective drugs. Two hundred microliter aliquots were taken from the medium hourly up to 4 hours and mixed with trichloroacetic acid (TCA) to a final concentration of 10%. The resulting mixture was centrifuged at 20,000 g and was carefully decanted. The supernatant, which contains the TCA-soluble radioactive amino acids and peptides is processed for liquid scintillation counting. At the end of 4 hours, the medium is completely removed and the cells are lysed with 2mL of 0.2 N NaOH. One hundred microliters of the resulting lysate is taken and is processed for liquid scintillation counting. Liquid scintillation counting was done using Wallac 1450 MicroBeta Trilux liquid scintillation & luminescence counter (PerkinElmer). The degradation rate was calculated according to the scheme described in the original protocol (26).

### Proteasome Extraction and Isolation

HEK293 Rpn11-HTBH cells were grown to 70-80% confluence and were exposed to 1µM b-AP15 or 0.1% DMSO. The cells were then lysed with buffer containing 50mM HEPES, 5mM MgCl_2_, 0.5% Triton X-100, 10% glycerol, 2 mM ATP, 50 mM NaCl and clarified by centrifugation. An amount of lysate equivalent to 2 mg of protein was mixed with 500 µg of Pierce™ Streptavidin Magnetic Beads (Thermofisher), diluted with wash buffer containing 50 mM HEPES, 5 mM MgCl_2_, 10% glycerol, 2 mM ATP, 50 mM NaCl, and was incubated overnight at 4°C. The beads were then separated with a magnetic rack, washed, and mixed with 0.1 U/µl AcTEV™ Protease (Thermofisher) in wash buffer and incubated for 1 h at 30°C. The supernatant, which contains the 26S proteasome, was then separated from the streptavidin beads.

### SDS-PAGE and Immunoblotting

Cells were lysed using RIPA buffer, and lysates were prepared for electrophoresis with NuPAGE™ sample reducing agent (Thermofisher) and NuPAGE™ SDS loading buffer (Thermofisher). Samples were heated to 95°C for 5 minutes. A total of 25 µg of protein was resolved with SDS-PAGE using 3-8% tris-acetate gels for blots probing polyubiquitin or 4-12% bis-tris gels for all the others. Proteins were transferred to nitrocellulose membranes using Thermo Scientific™ Pierce™ G2 Fast blot. Membranes were incubated in primary antibody diluted in PBST with 2% BSA overnight at 4°C, washed, and incubated with the appropriate secondary antibody diluted in PBST with 5% skimmed milk. Blots were developed using Clarity Western ECL substrate (Bio-Rad) and imaged using ChemiDoc MP Imager (Biorad). All images were processed in ImageLab v 5.2.1 (Biorad).

### Gene Expression Analysis

MCF-7 cells were treated for 6 hours with 1µM b-AP15 or DMSO. Cells were then lysed and RNA was isolated using the RNeasy Plus Mini Kit (Qiagen, Hilden, Germany). 500 nM of total RNA was further processed for microarray analysis using the GeneChip WT PLUS Reagent Kit according to manufacturer instructions (Affymetrix, Santa Clara, CA, USA). Prepared RNA was hybridized to GeneChip HuGene 2.0 ST chips for 16 h using the GeneChip Hybridization Oven 645, washed and stained using the GeneChip Fluidics Station 450 using the FS450_0002 protocol, and scanned with a GeneChip Scanner 3000 7G (Affymetrix). Data analysis was performed using R 4.0.2 and RStudio 1.3.1093. Unannotated probes were omitted, and differential expression was calculated. To identify differentially regulated genes, we used an adjusted p-value of <0.05 and a fold-change of >2 or <0.5. For duplicate probes, only the one with the lowest p-value was retained in the analysis. Gene set enrichment analysis was done on GSEA 4.03 (27) using a ranked list of genes based on the calculated t-statistic and Hallmarks and Gene Ontology as gene set databases. Network analysis was done using the EnrichmentMap and AutoAnnotate applications for Cytoscape 3.8.2. Enrichment map settings for Node Cutoff Q value <0.05, and Edge Cutoff < 0.5. Some of the clusters were annotated manually for clarity (28–30).

### MALDI-TOF

10 µg (7 µM) of recombinant USP14 was reacted with DMSO or 10 µM of b-AP15 at 30°C for 1 hour in 10 mM Tris-HCl pH 8.0. The samples were then desalted using 30 kDa cut-off Microcon-30 Centrifugal filters (Millipore) and reconstituted with deionized water. Approximately 0.25 µg of USP14 was loaded onto polished stainless steel MALDI-TOF plates with 20 µg sinapic acid (Sigma) in 70% acetonitrile/0.03% Formic Acid solution and air-dried. Whole protein mass spectra were acquired on an UltrafleXtreme MALDI-TOF mass spectrometer (Bruker Daltonics) operated in the linear positive ion mode with flexControl software v 3.4. Each spectrum was the cumulative signal from approximately 5000 laser shots. The MS spectra were externally calibrated using the Protein Calibration Standard II mixture (Bruker Daltonics). The MS spectra were analyzed using flexAnalysis v 3.4 (Bruker Daltonics).

### LC-MS/MS and Label-Free Quantification

The samples were reduced by incubating with 5 mM dithiothreitol (DTT) at 56°C for 25 min and subsequently alkylated with 15 mM of iodoacetamide (IAA) at room temperature for 30 min. Excess IAA was quenched by the addition of DTT to 10 mM. The samples were subjected to methanol-chloroform precipitation and the obtained protein pellet was washed with methanol once and dried in a SpeedVac (Savant, Thermo) vacuum concentrator. The samples are then digested with 0.35 µg/µL of Pierce MS grade trypsin protease (Thermo Scientific) in 50 mM NH_4_HCO_3_ at 37°C for 16 h. The resulting peptide digests were then dried in a vacuum concentrator, reconstituted with 0.1% trifluoroacetic acid, and analyzed using LTQ Orbitrap Velos Pro mass spectrometer (Thermo) coupled to an EASY-nLC II (Thermo Scientific) chromatography system. Peptides were separated by reverse-phase chromatography using a 20 mm x 100 µm C18 precolumn followed by a 100 mm x 75 µm C18 column with a 5 µm particle size (NanoSeparations) using the following solvent system: Buffer A, 0.1% Formic Acid v/v in water; Buffer B, 0.1% Formic Acid v/v in Acetonitrile. The gradient starts with Buffer B at 2% to 40% in 90 min, then to 90% in 20 min, 90% Buffer B is maintained for 7 min and dropped to 2% in 3 min for a total elution time of 120 minutes. The mass spectrometer was operated using XCalibur software v2.6 (Thermo Scientific) and analysis was done in positive ionization mode. Full scans were performed at a resolution of 30,000. The top 20 most intense ions were fragmented with Collision-induced dissociation (CID) using a normalized collision energy of 35%, isolation width of 2.0, and activation time of 10 ms. Protein identification and quantification were done with Proteome Discoverer 2.5.0.400 (Thermo) using the Sequest HT search engine. All searches were performed on human reference proteome obtained from UniProt (http://www.uniprot.org Accessed: 7 November 2020). Cysteine carbamidomethylation was set as a fixed modification and methionine oxidation as a variable modification. For the experiments on recombinant USP14 protein, carbamidomethylation and b-AP15 were set as dynamic modifications. Label-free quantification of proteins was done and protein abundances were quantified based on precursor intensity. The abundances were then normalized based on the total peptide amount. Proteins that were identified with high confidence, had at least 2 peptides, and were present in at least 2 of 3 samples in both groups were considered for quantification. Proteins considered significantly changed have a log-fold change of >1 or <-1 and p-value of <0.05 on a Student’s T-test. Paired T-tests were done on select protein groups. All statistical analyses were performed on Microsoft Excel.

### Genome-Wide CRISPR/Cas9 Knockout Library Screen

Generation of stable Cas9 expressing cells. HCT116 cells were grown in McCoy’s 5a supplemented with GlutaMAX™ (ThermoFisher), 10% fetal bovine serum (Gibco), and penicillin-streptomycin (Gibco). Parental cells were lentivirally transduced with pLenti-Cas9-T2A-Blast-BFP to express a codon-optimized WT SpCas9 flanked by two nuclear localization signals linked to a blasticidin-S-deaminase – mTagBFP fusion protein *via* a self-cleaving peptide (derived from lenti-dCAS9-VP64_Blast, a gift from Feng Zhang, Addgene #61425). Following blasticidin selection, a stable BFP+ population was isolated by repeatedly sorting for BFP expressers.

#### Guide Library

The genome-wide Brunello sgRNA library (31) was synthesized as 79 bp long oligos (CustomArray, Genscript). The oligo pool was double-stranded by PCR to include an A-U flip in the tracrRNA (32), 10 nucleotide long random sequence labels (RSLs), and an i7 sequencing primer binding site (33).The resulting PCR product (array oligo in bold) with the sequence ggctttatatatcttgtggaaaggacgaaacaccgnnnnnnnnnnnnnnnnnnnngtttaagagctagaaatagcaagtttaaataaggctagtccgttatcaacttgaaaaagtggcaccgagtcggtgcttttttGATCGGAAGAGCACACGTCTGAACTCCAGTCACNNNNNNNNNNaagcttggcgtaactagatcttgagacaaa was cloned by Gibson assembly into pLenti-Puro-AU-flip-3xBsmBI (33). The plasmid library was input sequenced to confirm representation and packaged into lentivirus in HEK-293T (ATCC) using plasmids psPAX2 (a gift from Didier Trono, Addgene #12260) and pCMV-VSV-G (a gift from Bob Weinberg, Addgene #8454). The virus-containing supernatant was concentrated with Lenti-X concentrator (Takara), aliquoted, and stored in liquid nitrogen.

#### Library Virus Titration and Large-Scale Transductiony

The functional titer of the library virus was estimated from the fraction of surviving cells after the transduction of target cells with different amounts of virus and puromycin selection. For the screen, Cas9-BFP-expressing target cells were transduced with the library virus in duplicate at an approximate MOI of 0.3 and a coverage of 1,000x (1,000 cells per guide) in the presence of 2 µg/ml polybrene. Transduced cells were selected with 2 µg/ml puromycin from day 2 to day 6 post-transduction. A control sample worth 80 million cells per replicate were harvested at day 4 post-transduction. Cell numbers per replicate were kept at >= 80 million/replicate throughout to ensure full library coverage.

#### Genomic DNA, Library Preparation, and NGS Sequencing

Genomic DNA was isolated using the QIAamp DNA Blood Maxi Kit (Qiagen), and guide and UMI sequences were amplified by PCR as previously described (33), using modified primers PCR2_FW acactctttccctacacgacgctcttccgatctcttgtggaaaggacgaaacac and PCR3_fw aatgatacggcgaccaccgagatctacac [i5] acactctttccctacacgacgctct, respectively. The amplicon was sequenced on Illumina NovaSeq, reading 20 cycles Read 1 with custom primer CGATCTCTTGTGGAAAGGACGAAACACCG;10 cycles Index read i7 to read the UMI, and six cycles Index read i5 for the sample barcode. NGS data was analyzed with the MaGeCK software (34) and by UMI lineage dropout analysis (33).

### PISA Analysis

#### PISA in Lysate

PISA in living cells and cell lysate was performed as described previously (35). Cells were detached by trypsin (TrypLE, Gibco), washed twice with PBS (Gibco), and resuspended in PBS. The cell suspensions were freeze-thawed in liquid nitrogen 5 times and then centrifuged at 10,000 g for 10 min to remove the cell debris. The remaining lysate was aliquoted into 3 replicates and treated at 2.5 µM of b-AP15 or vehicle (DMSO) for 30 min at 37°C in 300 μL reaction volumes. Next, 300 µL was aliquoted row-wise in 10 wells of a 96-well plate and heated with a gradient ranging from 48-59°C for 3 min in an Eppendorf gradient thermocycler (Mastercycler X50s). Following a 3 min incubation at room temperature, samples were snap-frozen with liquid nitrogen and kept on ice. To remove the insoluble fraction, the 10 aliquots of each replicate were combined and 220 µL was transferred into thickwall polycarbonate tubes (Beckman Coulter, USA) and centrifuged at 100,000 g for 20 min in a Beckman Coulter Optima XPN-80 ultracentrifuge.

After centrifugation the supernatant was transferred into new Eppendorf tubes, leaving the insoluble pellet behind. The protein concentration of each sample was measured using a Pierce BCA Protein Assay Kit (Thermo) and samples were normalized to 25 µg of protein in ~100 µL volume. DTT was added in each sample to a final concentration of 5 mM and incubated for 1 h at 25°C. Following DTT, a volume corresponding to a 15 mM final concentration of IAA was added and samples were incubated at 25°C in the dark for 1 h. Excess IAA was quenched by the addition of DTT to 10 mM. Proteins were then precipitated by methanol/chloroform. Samples were left to dry before being dissolved in 20 mM EPPS (pH=8.5) containing 8 M urea. Next, samples were diluted to 4 M urea using 20 mM EPPS (pH=8.5) before adding Lysyl endopeptidase (LysC; Wako) at 1:100 w/w (enzyme: protein) ratio for 6 h at 25°C. Samples were diluted again with 20 mM EPPS to the final urea concentration of 1 M and Trypsin (Promega) was added at a 1:100 w/w ratio and incubated for 6 h at 25°C. Acetonitrile (ACN) was added to a final concentration of 20% and a 4x w/w ratio of Tandem Mass Tags (TMTpro, Thermo) reagents were added to each sample and incubated for 2 h at 25°C. The reaction was quenched by adding hydroxylamine to a final concentration of 0.5%. Samples of each replicate were combined and dried for 1 h using a DNA 120 SpeedVac Concentrator (Thermo) until the ACN content had evaporated. Next, replicates were acidified by TFA, desalted with Sep-Pak C18 columns (Waters, USA), and dried overnight using DNA120 SpeedVac Concentrator (Thermo). The dried samples were resuspended in 20 mM ammonium hydroxide and fractionated using a Dionex Ultimate 3000 2DLC (Thermo) and XBrigde BEH C18 2.1x150 mm column (Waters; Cat#186003023). The method for fractionation includes a 48 min gradient from 1-63% B buffer (B=20 mM ammonium hydroxide in acetonitrile) in increments of three (1-23.5% B in 42 min, 23.5-54% B in 9 min, and then 54-63%B in 2 min) at 200 µL min^-1^ flow. Fractions were then concatenated into 16 samples in the following order (*e.g.* 1,17,33,49,65,81).

#### PISA in Cells

Cells were cultured until 70% confluence in 25 cm^2^ flasks and treated with b-AP15 at a concentration of 2.5 µM or with vehicle (DMSO) for 3 h at 37°C. The media was removed, cells were washed with PBS and 350 µL of PBS was added before scraping the cells. Cells were pipetted before aliquoting into 10 wells in PCR plates as in PISA analysis in cell lysate. The cells were then heated and cooled the same way as in PISA in cell lysate and then snap-frozen and kept on ice. Samples within each replicate were pooled and lysed by freeze-thawing 5 times using liquid nitrogen. The rest of the protocol for PISA in cells is the same as PISA in lysate.

The concatenated fractions were dissolved in buffer A (98%, H_2_O, 2% ACN, 0.1% FA) and loaded in PepMap C18 HPLC column (100 pore size, 75 µm diameter, Thermo) packed with an EASY-Spray column (Thermo, Cat#ES803A) and injected *via* UltiMate™ 3000 RSLCnano System (Thermo Fischer Scientific). Peptide elution was performed with buffer B (98% ACN, 2% H_2_0, 0.1% FA) during a 120 min optimized gradient. A Thermo Scientific Orbitrap Q-Exactive HF system was used to acquire mass spectra in data-dependent manner with automatic switching between MS and MS/MS modes with a maximum of 17 peptides per cycle starting from the most abundant ion (top-17 method). MS spectra were acquired at a resolution of 120 000 with a target value of 3E6 or a maximum integration time of 100 ms and the m/z range was set from 350 to 1500. Peptide fragmentation was performed using higher-energy collision dissociation at 33%. The MS/MS spectra were acquired at a resolution of 60 000 with the target value of 2E5 ions and a maximum integration time of 100 ms. The isolation window was set to 1.6 m/z units and the first fixed mass to m/z 100. Dynamic exclusion was 45s. LC-MS/MS files were searched in MaxQuant (v. 1.6.2.3) using UniProt mouse protein database excluding protein isoforms (55 228 entries). Cysteine carbamidomethylation was used as a fixed modification, while methionine oxidation, protein N-terminal acetylation, and deamidation of arginine and asparagine were selected as variable modifications. Trypsin/P was selected as enzyme specificity. No more than two missed cleavages were allowed. A 1% false discovery rate was used as a filter at both protein and peptide levels. First search tolerance was 20 ppm (default), and main search tolerance was 4.5 ppm (default), and the minimum peptide length was 7 residues. Match between runs was activated with a match time window of 0.7 min and an alignment time window of 20 min.

#### Data Availability

PISA data have been deposited to ProteomeXchange Consortium (http://proteomecentral.proteomexchange.org) *via* the PRIDE partner repository with data set identifier PXD028398.

## Results

### Gene Expression Profiling Indicates Effects of b-AP15 on the UPS

**Figure 2:**
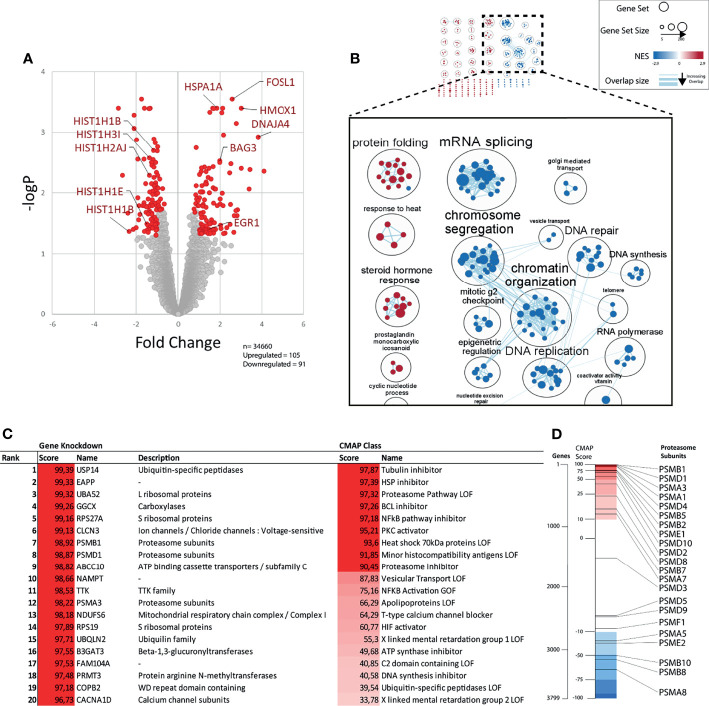
Gene expression profile after b-AP15 treatment. MCF7 cells were treated for 6h with either 1µM of b-AP15 or DMSO. Relative change in gene expression was obtained through microarray analysis. **(A)** Of the 34660 probes, 196 were differentially expressed. 105 were upregulated and 91 were downregulated in the relative to the control (adjusted p value < 0.05; more than 2-fold up or down regulated; n=3). Each point represents a probe. Immediate early genes (EGR1, FOSL1), HMOX1, chaperones (HSPA1A, BAG3, DNAJA4), and downregulated histones are shown. **(B)** Network analysis of enriched gene sets. Enriched gene sets (FDR < 0.05) were clustered based on their degree of overlap (number of similar genes). Gene sets overrepresented in upregulated genes have a positive NES (red) and downregulated genes have negative NES (blue). The top figure shows all the clusters. The magnified set at the bottom shows the select related clusters **(C)** The top differentially expressed genes were analyzed using the connectivity map (CMap) to find gene knockdowns or CMap classes in the CMap database that have a similar gene expression signature. Scores >90 are considered significant. **(D)** Bars show the position of curated gene knockdown signatures (black lines) relative to all 3799 gene knockdown signatures (spectrum) in the CMap database. Positive scores (red region) represent the correlated and negative scores (blue region) represents the inversely correlated knock-down gene signatures.

The cytotoxicity of b-AP15 and related dienone compounds has been attributed to different mechanisms. To determine the primary response to b-AP15 treatment at the mRNA level, MCF-7 cells were exposed to 1 µM b-AP15 or vehicle for 6 hours, followed by RNA extraction and processing for microarray analysis. We observed a total of 196 differentially expressed genes from 34,660 unique probes (**Figure 2A**). Upregulated genes included chaperones, intermediate early genes such as *FOSL, FOSB*, and *EGR1*, and the oxidative stress marker *HMOX*. Downregulated genes included histone genes and *CDK1* (**Figure 2A**). We analyzed for gene set enrichment against the Hallmarks and Gene Ontology gene set databases. Of the 5,300 gene sets in the database, 326 were significantly enriched (FDR < 0.05) (**Supplementary Table 1**). For the upregulated genes, 4 of the 10 most over-represented gene sets involved response to heat shock and protein refolding. For the downregulated genes, the top gene sets mainly involved nucleosome assembly and transcription regulation. Many of these gene sets have a high degree of overlap and so were clustered based on similarity and then annotated following a previously described workflow (36). Some gene set clusters were annotated manually for clarity (**Figure 2B** and **Supplementary Table 1**). The largest clusters for upregulated genes included gene sets on protein folding, steroid hormone response, and cardiac morphogenesis (**Supplementary Figure 1**). The clusters for downregulated genes form a large network involving mRNA splicing, chromatin organization and transcription, and G2-M cell cycle phase arrest.

We performed connectivity map (CMap) analysis (37) to find gene expression signatures that show connectivity with the gene expression induced by b-AP15 (**Figures 2C, D** and **Supplementary Table 2**). The analysis was limited to knockdown signatures and Cmap classes. Among the knockdown signatures, the proteasome-associated gene *USP14* showed the strongest connectivity, consistent with the known inhibitory action of b-AP15 on this DUB (5). Knockdown of the expression of the genes encoding the integral proteasome subunits PSMA1, PSMA3, PSMB1, and PSMD4 also showed strong connectivity to the b-AP15 signature. Ten of the 22 proteasome subunit knockdown signatures rank within the top 10% that show the highest connectivity to the b-AP15 signature **(** **Figure 2D** **)**. Among the Cmap classes, tubulin inhibition showed the strongest connectivity. b-AP15 is known to arrest cells at G2/M due to the induction of cell cycle inhibitors (15, 38) which is likely to explain the similarity to the tubulin inhibitor signature.

### b-AP15 Inhibits Proteasomal Degradation

**Figure 3:**
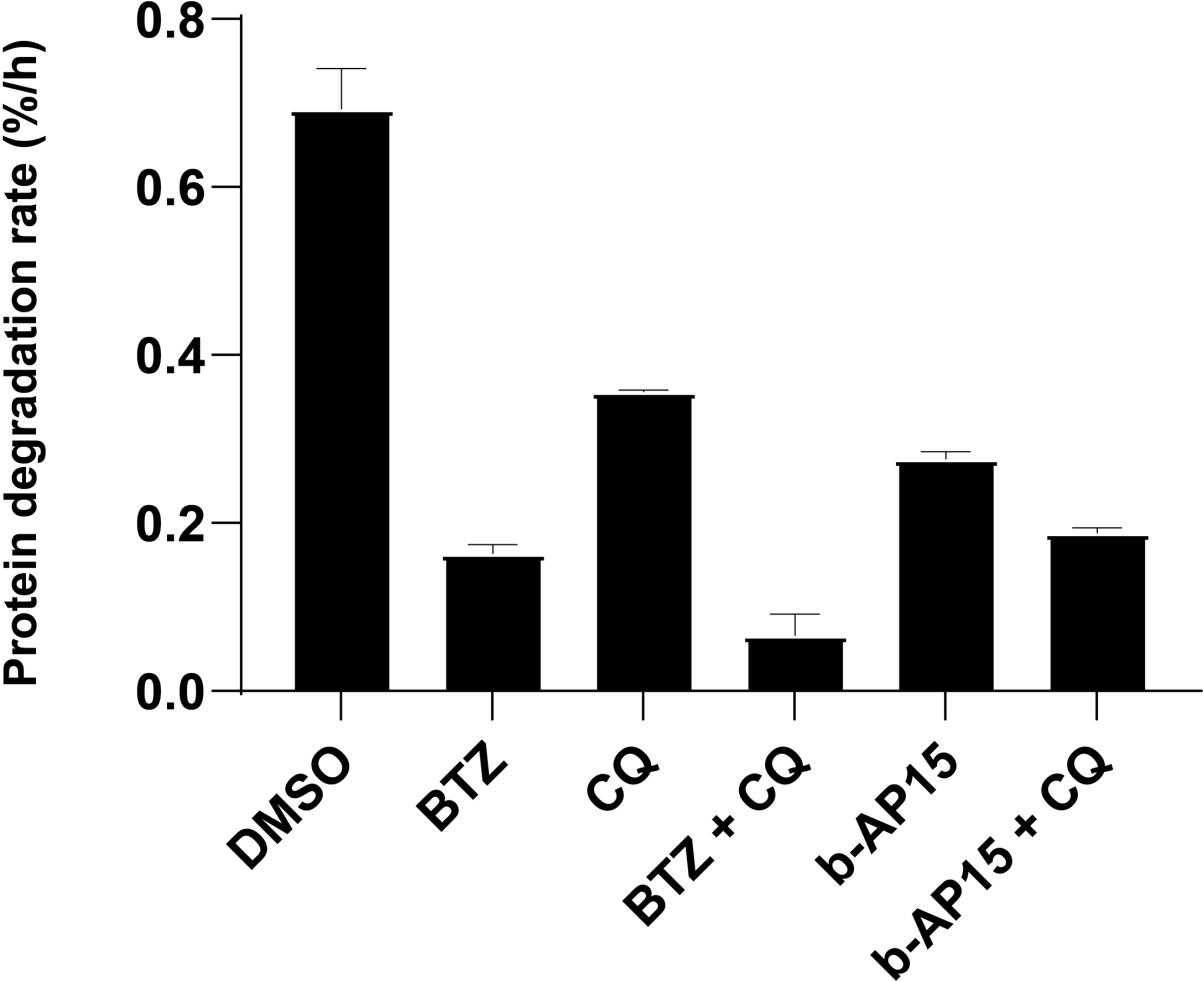
b-AP15 inhibits proteasomal degradation. Degradation of long-lived proteins. HCT116 cells were pulse-labeled with ^3^H-Phenylalanine and treated with the indicated drug or drug combination. The protein degradation rate is calculated from the acid-soluble radioactivity released into the media over 4 hours. The concentrations used are as follows: b-AP15 - 1µM, bortezomib (BTZ) - 1µM, chloroquine (CQ) – 40µM. Bars represent the mean of four replicates +/- SD; n=4. Pairwise comparisons except for BTZ vs b-AP15 + CQ (p = 0.77) are all significantly different (p < 0.05).

We and others have previously shown that b-AP15 and similar compounds induce the accumulation of proteasome substrates in cells [see (39)]. To examine the effects of b-AP15 on protein turnover, we measured the degradation rate of long-lived proteins in the presence of b-AP15 or bortezomib, as single agents or in combination with the autophagy inhibitor chloroquine, through a pulse-chase radiolabeling assay (26). We limited the analysis to long-lived proteins as the mean degradation rate remains linear for up to 10 hours and is straightforward to interpret (26). Protein degradation was reduced by both bortezomib (~ 76% reduction) and b-AP15 (~ 60% reduction) and was further reduced by the addition of chloroquine (~13-14% additional reduction) (**Figure 3**). Bortezomib is expected to abrogate the chymotrypsin and caspase-like (β1 and β5 subunits), but not the trypsin-like (β2), activity of the 20S proteasome subunit, which may explain the residual degradation observed (40). Interestingly, treatment with chloroquine alone results in ~ 50% reduction in protein degradation, which is greater than the additional reduction it confers when in combination with bortezomib or b-AP15 (**Figure 3**). This discrepancy could be because chloroquine has some inhibitory activity on the 20S proteasome (41). Previous studies have shown that b-AP15 enhances autophagic flux (13, 17), thus it is unlikely that the decrease in protein turnover is due to inhibition of autophagy. These results confirm and strengthen previous observations that b-AP15 inhibits proteasomal degradation.

### Effect of b-AP15 Treatment on the Proteasome and Proteasome-Associated Proteins

**Figure 4:**
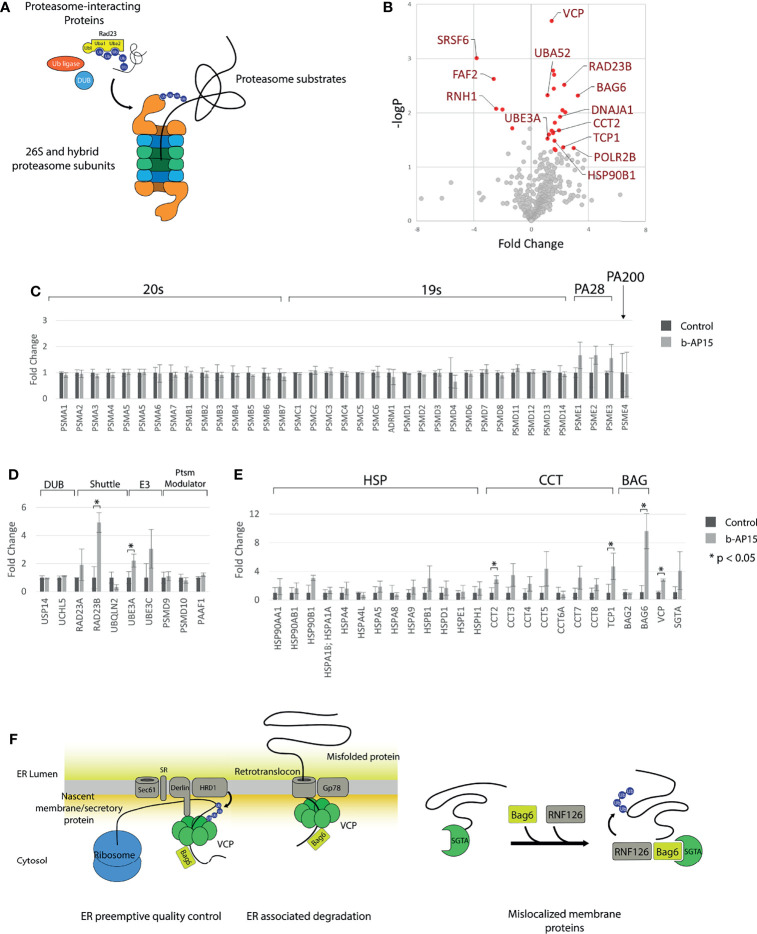
Quantification of proteasome and proteasome-associated proteins. Proteasomes and proteasome-associated proteins were affinity purified from HEK293-Bio-Rpn11 treated with either DMSO or 1µM b-AP15 and processed for LC-MS/MS and label-free quantification. **(A)** Types of proteins pulled down with HBTH-tagged proteasomes **(B)** 653 of the 757 identified proteins could be quantified. Cutoff values for significantly different proteins are logFCs > 1 or < and p-value < 0.05 **(C)** Relative amounts of 20S core particle and the 19S core particle. PA28 subunits are collectively increased with b-AP15 treatment (paired t-test p = 0.002) **(D)** Relative amounts of proteasome interacting proteins are largely unchanged. The levels of the Uba-Ubl protein RAD23B and the E3 ligase UBE3A is increased with b-AP15 treatment. **(E)** Heat shock proteins and components of the TRiC/CCT complex are collectively increased (paired t-test p < 0.002 for both). **(F)** VCP, BAG6 and SGTA relay protein substrates to the proteasome in the different protein quality control processes. b-AP15 treatment results in stalling of the proteasome and co-purification of these proteins. *p < 0.05.

To determine the effect of b-AP15 on proteasome composition and the association of proteasome interacting proteins, we performed mass spectrometry-based quantification of proteasomal proteins that had been affinity-purified from b-AP15- or vehicle-treated HEK293-Bio-Rpn11 cells. These cells express an HBTH-tagged PSMD14 that facilitates proteasome purification (42). The 757 identified proteins generally belonged to 20S proteasome core particle, 19S/PA28/PA200 regulatory particle subunits, proteasome-interacting proteins, and proteasome substrates (**Figure 4A** **).** Out of 653 quantified proteins, 22 were significantly increased and five were significantly decreased in the b-AP15-treated sample (**Figure 4B** and **Supplementary Table 3**). The levels of 20S and 19S regulatory particle subunits were similar but the quantities of PSME1, PSME2, and PSME3, components of PA28αβ and the PA28γ regulatory particles, were increased in the b-AP15-treated cells. This could indicate increased formation of hybrid proteasomes as a stress response mechanism (**Figure 4C**). Formation of this type of proteasome complexes has been shown to occur in response to proteasome inhibition, possibly in order to facilitate degradation of misfolded proteins during conditions of proteotoxic stress (43). Among the proteasome interacting proteins, RAD23B was increased >4-fold and UBE3A was increased 2-fold (**Figure 4D** **)**. RAD23B is known to shuttle polyubiquitinated proteins to the proteasome (44). We also found that ubiquitin (identified as the ubiquitin fusion protein Uba52) was increased 2-fold (**Figure 4B**). Heat shock proteins and components of the CCT/TriC chaperonin complex were collectively increased in samples from b-AP15 treated cells (**Figure 4E**). We found increases in VCP/p97/Cdc48 (>2-fold) and BAG6 (>9-fold), chaperones that couple the proteasome to ER-associated degradation (ERAD) and to the ER-preemptive quality control pathway for nascent proteins. SGTA (small glutamine-rich tetratricopeptide repeat-containing protein alpha), which together with BAG6 facilitates the degradation of tail-anchored proteins and mislocalized membrane proteins (MLP) (45), trends towards an increase (**Figure 4E**). These processes are illustrated in **Figure 4F**. The findings are congruent with the notion that there is an increased association of misfolded proteins with the proteasome due to proteasome inhibition and are consistent with previous observations of ER stress in b-AP15-exposed cells (46).

### b-AP15 Adducts USP14 at Cys203 and Cys257

**Figure 5:**
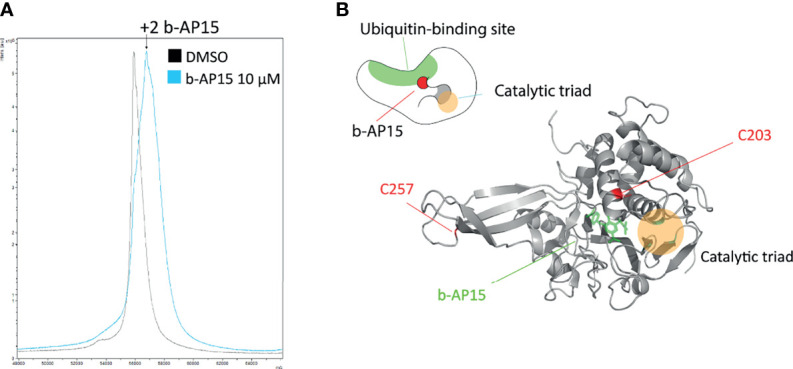
b-AP15 binds USP14. Recombinant USP14 was exposed to 10µM b-AP15 or DMSO for 1 hour and subjected to mass spectrometry to identify binding sites. **(A)** MALDI-TOF mass spectrum Peak from b-AP15-treated sample shows a mass shift of roughly 810 Da corresponding to 2 molecules of b-AP15. LC-MS/MS shows b-AP15 modifications on Cys203 and Cys257 (**Supplementary Figure 2** **) (B)** Docking study of b-AP15 on crystal structure of USP14 (2ayo) reveals that b-AP15 docks on between the ubiquitin binding surface and the crevice that leads to the catalytic triad. Top drawing is a representation of b-AP15-bound USP14.

Previous findings have indicated transient association of b-AP15 analogues to the ubiquitin-binding crevice of the proteasome-associated DUB USP14, followed by slow irreversible binding (16). We exposed recombinant USP14 to b-AP15 and examined adduction using mass spectrometry. A mass shift of ~810 D was observed using MALDI-TOF (**Figure 5A**), consistent with the binding of two b-AP15 molecules (M.W. 419). We extended the analysis by using LC-MS/MS with the *a priori* assumption that dienones largely modify cysteine residues. Cys203 and Cys257 were identified as being adducted by b-AP15 (**Supplementary Figure 2**). We performed *in silico* docking of b-AP15 to the catalytic domain of USP14 [2AYO (47)] with AutoDock Vina (48, 49). The tentative b-AP15 binding pocket is located close to the active site that houses the C-terminal tail of ubiquitin in the Ub-loaded state (**Figure 5B**). The result is consistent with the previous finding of docking of USP14 inhibitors to this crevice (16) with Cys203 situated closer to the ubiquitin-binding site than the catalytic Cys114 (**Figure 5B**).

### Effects of USP14 and UCHL5 Deletion on the Response to b-AP15

**Figure 6:**
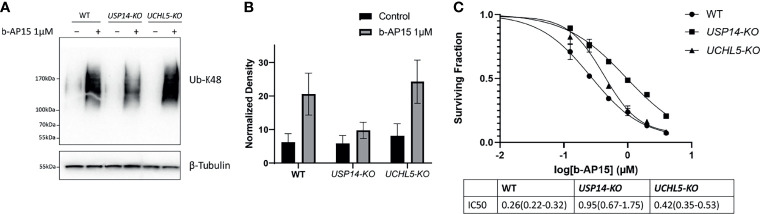
b-AP15 cytotoxicity is partially mediated by USP14. **(A)** Parental HCT116, *USP14-KO*, and *UCHL5-KO* were exposed to either b-AP15(1µM) or DMSO for 8 hours. Whole cell lysates were obtained and probed for K48 polyubiquitin or β-tubulin **(B)** K48 polyubiquitin smears normalized to the corresponding β-tubulin bands. Each bar is the average normalized density from three independent samples +/- SD. The differences between b-AP15 and DMSO groups across all cell-types are not statistically significant **(C)** The sensitivity of parental and mutant cell lines to b-AP15 was studied using the MTT assay. Points represent the mean of four replicates +/- SD. The estimated and 95% confidence interval for the IC50s are listed in the table below the plot.

We examined how the loss of USP14 or UCHL5 affects sensitivity to b-AP15 by generating knockout (KO) HCT116 cells for *USP14* or *UCHL5* using CRISPR/Cas9 (see *Materials and Methods*). Successful deletion was verified by PCR and immunoblotting (**Supplementary Figure 3**). Treatment with b-AP15 results in the accumulation of K48-linked polyubiquitinated proteins in all the cell lines. This effect was lower in *USP14-KO* cells compared with the control or *UCHL5-KO* cells (**Figures 6A, B** **)**. The sensitivity to b-AP15 was measured using the MTT cell viability assay (**Figure 6C**). An approximately 2-fold increase in the IC_50_ was observed for *USP14-KO* cells compared to parental cells whereas the sensitivity of *UCHL5-KO* cells to b-AP15 was only marginally affected (**Figure 6C**). Taken together, these results are consistent with the notion that USP14 inhibition is mechanistically important for the activity of b-AP15, but that other targets may also be involved.

### Identification of b-AP15 Targets Using Proteomics

**Figure 7:**
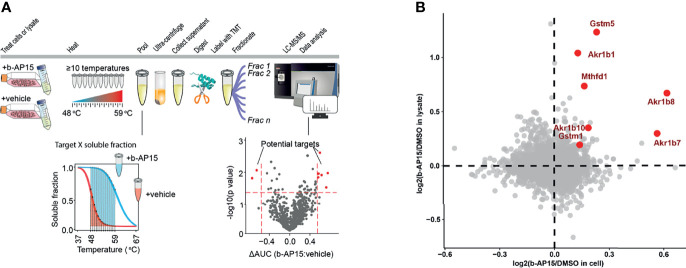
PISA analysis. **(A)** Schematic representation for PISA in living cells and cell lysate. B16-F10 cells or native lysate is treated with b-AP15 or vehicle (DMSO) and 10 aliquots are obtained. Each aliquot is heated to a specific temperature for 3 min. The aliquots corresponding to different temperature points are then pooled and centrifuged to collect the soluble fractions. The collected pools are then digested with trypsin, labeled with TMT and fractionated to extend the proteome coverage. The fractions are then subjected to LC-MS/MS and data is extracted and analyzed. The comparison of soluble fractions (area under the curve or AUC) for each protein in the presence and absence of the compound will highlight the drug targets. **(B)** The overlay of protein solubility in living cells vs. cell lysate highlights the proteins that are more soluble in the presence of b-AP15, indicating that they directly bind to the compound. *P*-values were calculated using a paired Student’s *t*-test (n=4). The highlighted proteins (red) have a *p*<0.05 in both living cell and lysate experiments.

We used Proteome Integral Solubility Alteration (PISA) (50), the high-throughput proteome-wide implementation of thermal proteome profiling (TPP) or MS-CETSA (51) to search for b-AP15 targets (**Figure 7A** **)**. Since the proteasome is a multi-subunit complex that precipitates during conditions of heating [discussed in (16)], PISA was not expected to identify individual proteasome components as targets. However, PISA analysis resulted in the identification of five proteins that were more soluble both in cell and lysate in the presence of the compound, indicating direct interaction with b-AP15 (**Figure 7B**). Three of these were aldo-ketoreductases, known to be involved in the reduction of carbonyl groups to primary and secondary alcohols on a wide range of substrates (52). The experiment also identified Gstm5 (glutathione-S-transferase Mu 5) as a target of b-AP15 (**Figure 7B**). Glutathione-S-transferases are known to be targeted by electrophilic compounds (53). The fifth protein identified by this analysis was methylenetetrahydrofolate dehydrogenase 1 (Mthfd1), an enzyme involved in one-carbon metabolism and described as a target for arsenic trioxide (54).

### Identification of Mechanisms of b-AP15 Sensitivity Using Genome-Wide CRISPR/Cas9 Library Screening

**Figure 8:**
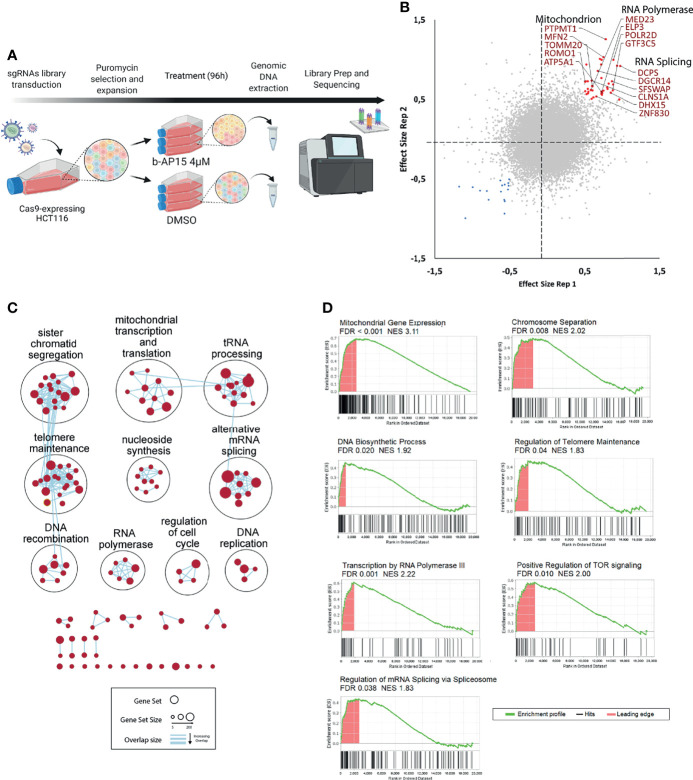
CRISPR-Cas9 loss-of-function screening. **(A)** Schematic representation of the strategy for the CRISPR dropout screen. Cas9-expressing HCT116 cells were transduced with an sgRNA library targeting 19,364 protein-coding genes. Transduced cells were selected using puromycin. The cells are then expanded, split into two groups and treated with either b-AP15 or DMSO for 96 hours. After the treatment, genomic DNA is extracted and sequenced. **(B)** Correlation plot for two replicates. Red points represent guides that were enriched (>0.5 effect size) and blue points are the guides that were depleted (<-0.5 effect size) in the b-AP15-treated vs control cells in duplicate samples. Effect size is the log 2 of the ratio of normalized read counts for the sgRNA in the b-AP15-treated vs control cells. Guides present in enriched gene sets (mitochondrial genes, RNA polymerase components, and genes involved in RNA splicing) are labeled. **(C)** Network analysis of enriched gene sets. Gene set enrichment analysis was done on the sgRNAs ranked from the most enriched (targeting genes that confer sensitivity) to the most depleted (targeting genes that confer resistance). All gene sets with FDR < 0.1 were clustered based on their degree of overlap or similarity in genes. No gene sets were significantly overrepresented for depleted sgRNAs. **(D)** Enrichment of representative gene sets from the identified gene set clusters. The bar below the enrichment plot show the order of genes belonging to the gene set (hits) in the list of sgRNAs ranked from the most enriched (left) to the most depleted (right). Leading edge are the hits that contribute most to the gene set enrichment score.

Screening of CRISPR/Cas9 knock-out libraries can be used for the identification of genes that are important for drug sensitivity (55). We utilized a library that contains 77,441 unique sgRNAs, which targets 19,364 protein-coding genes to transduce Cas9-expressing HCT116 cells. The mutant cell pool was exposed to 4 μM b-AP15 or vehicle for 96 hours leading to ~70% cell death (**Figure 8A**). We identified 59 genes that affected the sensitivity to b-AP15 in duplicate samples (**Figure 8B** and **Supplementary Table 4**). Of these, 42 gene knock-outs resulted in decreased sensitivity to b-AP15 and 17 resulted in increased sensitivity. To determine if there are cellular processes or groups of genes that influence sensitivity to b-AP15, gene set enrichment and network analysis were done (**Figures 8C, D** **)**. Genes associated with resistance encoded for different classes of proteins. Deletion of mitochondrial genes was strongly associated with decreased sensitivity to b-AP15. This finding is consistent with previous results showing that the strong oxidative stress that is observed in b-AP15-exposed cells is coupled to mitochondrial damage (17, 23). Genes encoding proteins involved in mRNA splicing and associated with RNA polymerase activity were also identified (**Figure 8D** **)**.

## Discussion

Dienone compounds with a 1,5-diaryl-3-oxo-1,4-pentadienyl pharmacophore are electrophiles that primarily react with thiols and are predicted to engage multiple targets in cells. These compounds have, however, been described by a number of laboratories to display selective cytotoxicity to tumor cells [reviewed in (39, 56)] implying that a dominant pharmacological response is elicited. Previous reports have linked the cytotoxicity of dienones to inhibition of the UPS (5, 39, 57) but other effects such as impairment of translational elongation (58), altered conformation of the p53 tumor suppressor protein (59), inhibition of thioredoxin reductase (20, 60), and formation of protein complexes (24) have also been described. Our analysis using transcriptional profiling is consistent with the notion of a complex response to b-AP15, involving effects on protein folding but also mRNA splicing and chromosome segregation. Many cancers exhibit aberrant pre-mRNA splicing and deregulation of spliceosome components (61). b-AP15 is known to inhibit the cell cycle at the level of G2-M, consistent with the gene expression analysis (5, 15). The findings suggest that exposure to b-AP15 induces complex responses in cells, consistent with the expected polypharmacology of this type of compound.

b-AP15-induced cell death is strongly associated with the accumulation of a proteasome-degraded reporter in cells [see (39)]. We here extended previous findings by demonstrating that treatment with b-AP15 reduces the degradation of long-lived proteins. We also examined the effects of b-AP15 on the proteasome and on proteasome-associated proteins. The make-up of proteasomes was largely unaffected by b-AP15 exposure with the exception of increases in immunoproteasome subunits. PSME1, PSME2, and PSME3, components of PA28αβ and the PA28γ regulatory particles, are collectively increased which could indicate increased formation of hybrid proteasomes (**Figure 4C**). Formation of alternative proteasome complexes has been shown to occur in response to oxidative and electrophilic stress (62). Strong increases in the association of BAG6 (BCL2 Associated Athanogene 6) and SGTA (small glutamine-rich tetratricopeptide repeat-containing protein alpha) with proteasomes were observed. These are known constituents of the quality control system for hydrophobic proteins exposed to the cytoplasm (45). The association of the polyubiquitin shuttling factor RAD23B with proteasomes was increased >4-fold (**Figure 4D** **)**. This could reflect an increase in the delivery of ubiquitinated substrates or, alternatively, a consequence of proteasome stalling wherein RAD23 remains bound to the inhibited proteasome. Increased proteasomal association of the VCP/p97/Cdc48 ATPase was observed, as well as increased association with Hsp70, Hsp90, and T-complex protein Ring Complex (TriC) chaperones. The TriC is involved in the folding of approximately 10% of the proteome, including actins and tubulins (63). The pattern of alterations in proteasome-associated proteins is consistent with increased association of unfolded proteins with proteasomes and with ER stress consistent with previous data (46).

Inhibition of proteasomal activity by compounds containing the 1,5-diaryl-3-oxo-1,4-pentadienyl has been attributed to targeting of DUBs (5, 14, 20, 57, 64, 65). The transcriptional profile elicited by b-AP15 was found to be most similar to the profile induced by knock-down of the proteasome-associated deubiquitinase USP14 (**Figure 2C**). Our *a priori* hypothesis was that b-AP15 would engage the catalytic residue Cys114 of USP14 due to the low pK_a_ of this residue in the catalytic triad (47). However, we instead observed covalent binding of b-AP15 to Cys203 and Cys257. These residues lie within the ubiquitin-binding pocket of USP14. We examined the sensitivity of *USP14-KO* HCT116 cells to b-AP15 and observed a decreased sensitivity compared to parental cells. This effect was, however, limited and polyubiquitin accumulation was observed also in *USP14-KO* cells. The result shows that other components of the proteasome are targets for b-AP15. One such possible target is UCHL5, previously shown to bind the dienone compound RA-190 (66). The finding that *UCHL5-KO* cells showed similar sensitivity to b-AP15 as parental cells did not, however, support a role for this enzyme in conferring sensitivity. We conclude that proteasome-associated deubiquitinases can not be the sole targets determining sensitivity to b-AP15 or, with all probability, to other dienone compounds.

We utilized CRISPR/Cas9 loss-of-function screening in an attempt to identify b-AP15 targets. No strong association with a single target was observed. Deletion of USP14 was not associated with b-AP15 resistance as we expected. Proteasome inhibition and cell death ensues through processes independent of these DUBs. It is possible that the effect of deleting each contributing target is not large enough to be detected in the screen. We did find, however, that deletion of mitochondrial genes was associated with decreased sensitivity to b-AP15 (**Figure 8B**). b-AP15 is known to cause mitochondrial damage, possibly as a result of proteotoxic stress and the interaction of polyubiquitin complexes with the mitochondrial membrane (17). b-AP15 has also been shown to induce strong oxidative stress that contributes to the apoptotic response (46). Oxidative stress is strongly reduced in cells deprived of mitochondrial DNA (ρ0 cells) (23), strongly suggesting that mitochondrial damage results in the production of reactive oxygen species and cell death. We found that the sensitivity to b-AP15 was decreased by the deletion of genes encoding for components of the transcriptional and RNA maturation machinery and proteins involved in chromosome separation. These findings may be explained by more general effects on cell homeostasis resulting in decreased cell cycle progression and/or translation.

We used a proteomic method based on drug-induced alterations in protein solubility at elevated temperatures (PISA, Proteome Integral Solubility Alteration) (50) to identify b-AP15 target proteins. The proteasome is known to precipitate at 53°C (67). It is highly unlikely that the binding of a small molecule to one of the subunits of the proteasome will affect the melting temperature of the entire ~2.5 mDa complex and PISA was therefore not expected to identify proteasomal proteins or proteasome-associated proteins. We did not identify any deubiquitinases as b-AP15 targets using the PISA method despite findings of binding of dienones to USP1, USP18, and USP33, in addition to USP14 and UCHL5 (68). The PISA experiment, however, identified a number of other proteins as potential targets of b-AP15. Three of these were aldo-ketoreductases, known to be involved in the reduction of carbonyl groups to primary and secondary alcohols on a wide range of substrates (52). The experiment also identified Gstm5 (glutathione-S-transferase Mu 5). Glutathione-S-transferases are known to be targeted by electrophilic compounds (53). The fifth protein identified was methylenetetrahydrofolate dehydrogenase 1 (Mthfd1), an enzyme involved in one-carbon metabolism and described as a target for arsenic trioxide (54). Although these different proteins are not expected to be instrumental to the cytotoxic effects of b-AP15, the results demonstrate the potential of proteomic methods such as PISA in identifying drug targets.

Polypharmacology is commonly observed within the field of anticancer drugs, also with many targeted drugs (69). “Off-target” effects may indeed be essential for the anticancer activity of many drugs. Although b-AP15 and compounds containing the 1,5-diaryl-3-oxo-1,4-pentadienyl pharmacophore are capable of interacting with a number of cellular proteins, treatment using relevant concentrations results in a cellular response leading to selective tumor killing. The present study confirms previous studies regarding the role of the proteasome as an important target of b-AP15 but suggests that the mechanism of proteasome inhibition is complex and involves targets in addition to USP14.

Our study utilized three different methods for elucidation of the molecular mechanism of action and target identification. These different approaches resulted in quite different results: USP14 was the top hit using Cmap, detoxification enzymes were identified using proteomics, and mitochondrial proteins were implicated using loss-of-function screening. Our results nevertheless emphasize the need for a holistic approach using orthogonal methods for a deeper understanding of the mechanism of action of small molecules.

## Data Availability Statement

The datasets presented in this study can be found in online repositories. The names of the repository/repositories and accession number(s) can be found below: http://proteomecentral.proteomexchange.org/cgi/GetDataset PXD028398. Gene expression data has been deposited in GEO with the accession number GSE193542.

## Author Contributions

PD’A and SL, and JG supervised the project and conceived the experiments. JG and DKS carried out the experiments. PD’A, KS, and LS contributed to the preparation of samples. MT conducted tandem MS. BS and his group conducted the CRISPR/Cas9 loss-of-function screen. PS and RZ conducted PISA. PD’A, SL, JG, MT, PS, and BS analyzed and interpreted the data. All authors provided critical feedback that helped shape the project and this manuscript. All authors contributed to the article and approved the submitted version.

## Funding

This research was funded by Cancerfonden, Vetenskapsrådet (grant 2018-02570) and Radiumhemmets forskningsfonder. CFG acknowledges support from the National Genomics Infrastructure, SNIC (project 2017-7-265), and the Uppsala Multidisciplinary Center for Advanced Computational Science (UPPMAX). RZ acknowledges the Knut and Alice Wallenberg Foundation (grant KAW 2015.0063).

## Conflict of Interest

The authors declare that the research was conducted in the absence of any commercial or financial relationships that could be construed as a potential conflict of interest.

## Publisher’s Note

All claims expressed in this article are solely those of the authors and do not necessarily represent those of their affiliated organizations, or those of the publisher, the editors and the reviewers. Any product that may be evaluated in this article, or claim that may be made by its manufacturer, is not guaranteed or endorsed by the publisher.

## Supplementary Material

Supplementary Table 1 Gene set enrichment analysis results. POS and NEG sheets list gene sets with positive or negative enrichment scores (ES). Databases included in the search are Gene Ontology Biologic Process (BP) and Hallmarks.

Supplementary Table 2 cMAP analysis. List of all perturbagens in the cMAP database ranked according to connectivity to b-AP15 gene expression signature.

Supplementary Table 3 Mass spectrometry of proteasome-associated proteins. Quantified proteins that were present in significantly different amounts in control vs bAP15 treated samples.

Supplementary Table 4 Dropout screen. List of significantly enriched and depleted guides from two replicates. Guides that had >0.5 log fold change/effect size and < -0.5 log fold change/effect size in both replicates were considered enriched or depleted respectively.

Supplementary Figure 1 Network analysis of enriched gene sets from the gene expression dataset. Individual gene sets are represented by solid circles. Red for those overrepresented in upregulated genes and blue for downregulated genes. Size of the circles correspond to the size of the gene sets. Gene sets connected with a blue line have overlapping genes. Width of the line represents degree of overlap between gene sets.

Supplementary Figure 2 b-AP15 modification on C203 and C257. mass spectra of USP14 peptides identified to be modified by b-AP15 on MS/MS.

Supplementary Figure 3 Generation of DUB knockouts. **(A)** USP14 -/- cells were generated cells with floxed USP14. Transduction of cre recombinase results in the removal of the exon 5 and truncation of the USP14 protein. **(B)** Verification of USP14 exon 5 deletion by qPCR after cre transduction. **(C)** Presence of DUBs in the knockouts used. UCHL5 was knocked out through CRISPR/Cas9.

Supplementary Figure 4 Network analysis of enriched gene sets from the CRISPR dropout dataset. Individual gene sets are represented by solid circles. All gene sets presented here are overrepresented on guides that were enriched following bAP15 treatment. No gene set was significantly overrepresented on the depleted guides. The size of the circles correspond to the size of the gene sets. Gene sets connected with a blue line have overlapping genes. Width of the line represents degree of overlap between gene sets.
