# Supplementary figures and images for: Comprehensive Target Screening and Cellular Profiling of the Cancer-Active Compound b-AP15 Indicate Abrogation of Protein Homeostasis and Organelle Dysfunction as the Primary Mechanism of Action
Source: Front Oncol. 2022 Apr 22;12:852980. doi: 10.3389/fonc.2022.852980 (PMC9076133; doi:10.3389/fonc.2022.852980)

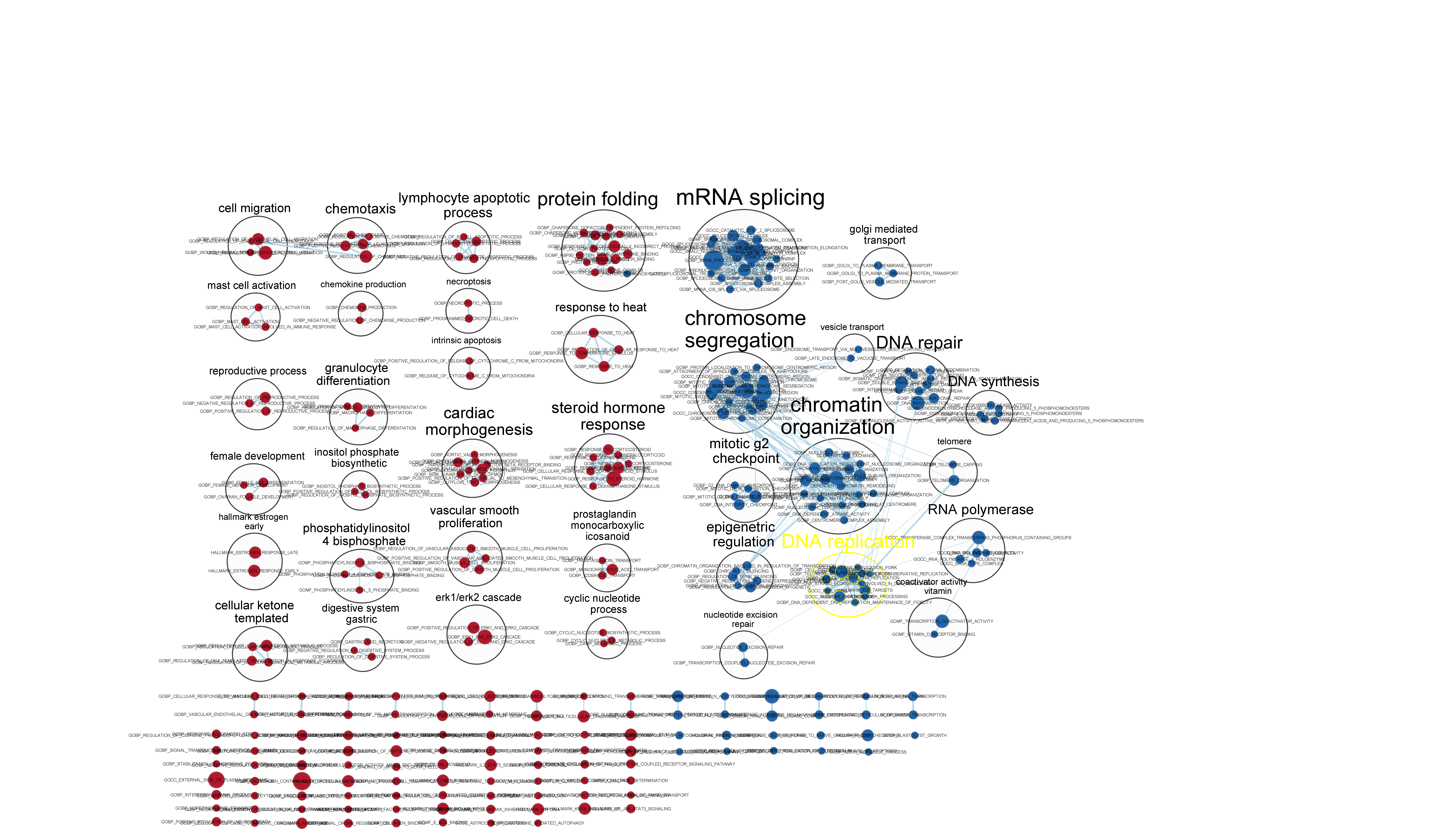

Supplement: Supplementary Figure 1 — Network analysis of enriched gene sets from the gene expression dataset. Individual gene sets are represented by solid circles. Red for those overrepresented in upregulated genes and blue for downregulated genes. Size of the circles correspond to the size of the gene sets. Gene sets connected with a blue line have overlapping genes. Width of the line represents degree of overlap between gene sets. [file Image_1.png]

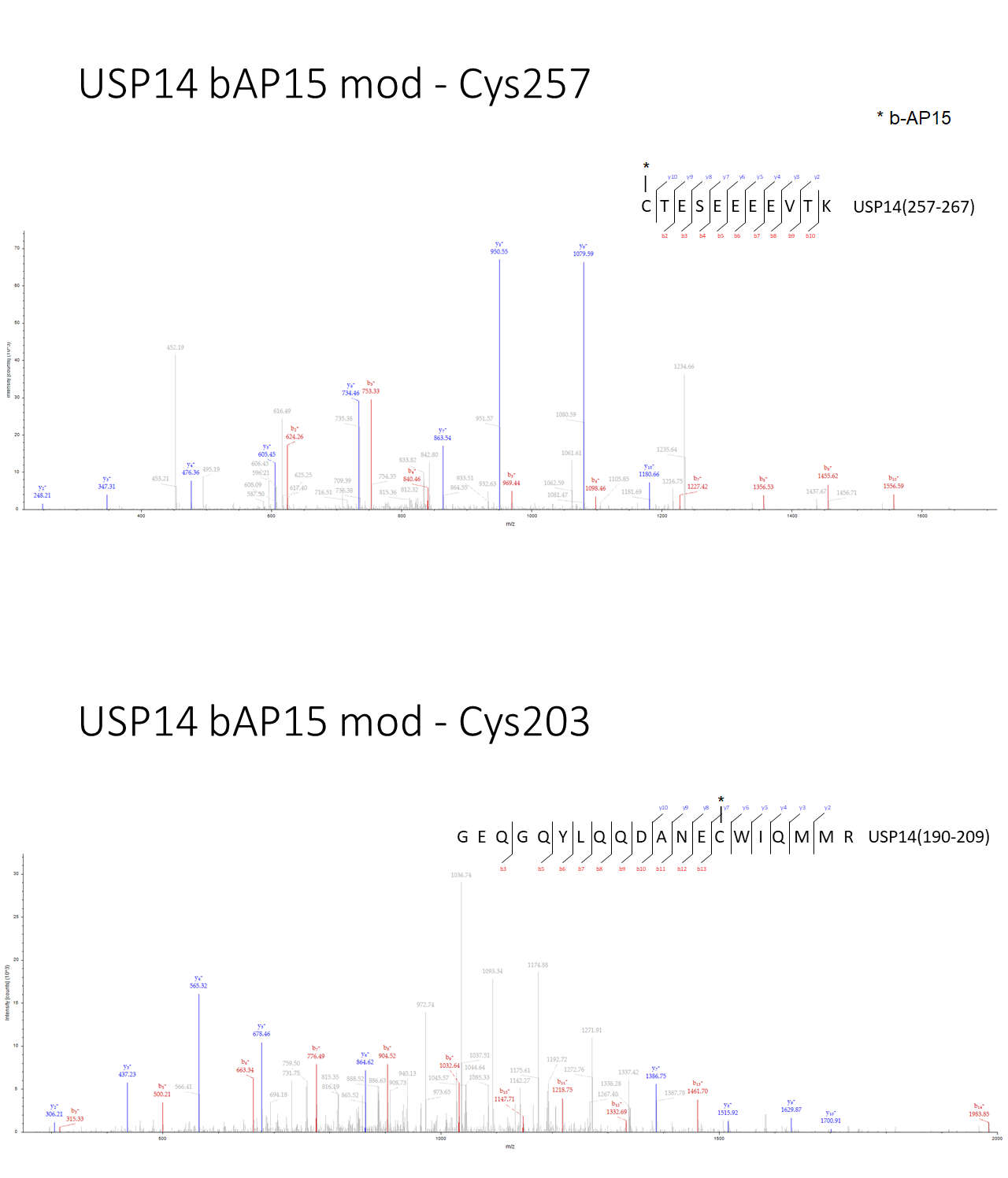

Supplement: Supplementary Figure 2 — b-AP15 modification on C203 and C257. mass spectra of USP14 peptides identified to be modified by b-AP15 on MS/MS. [file Image_2.png]

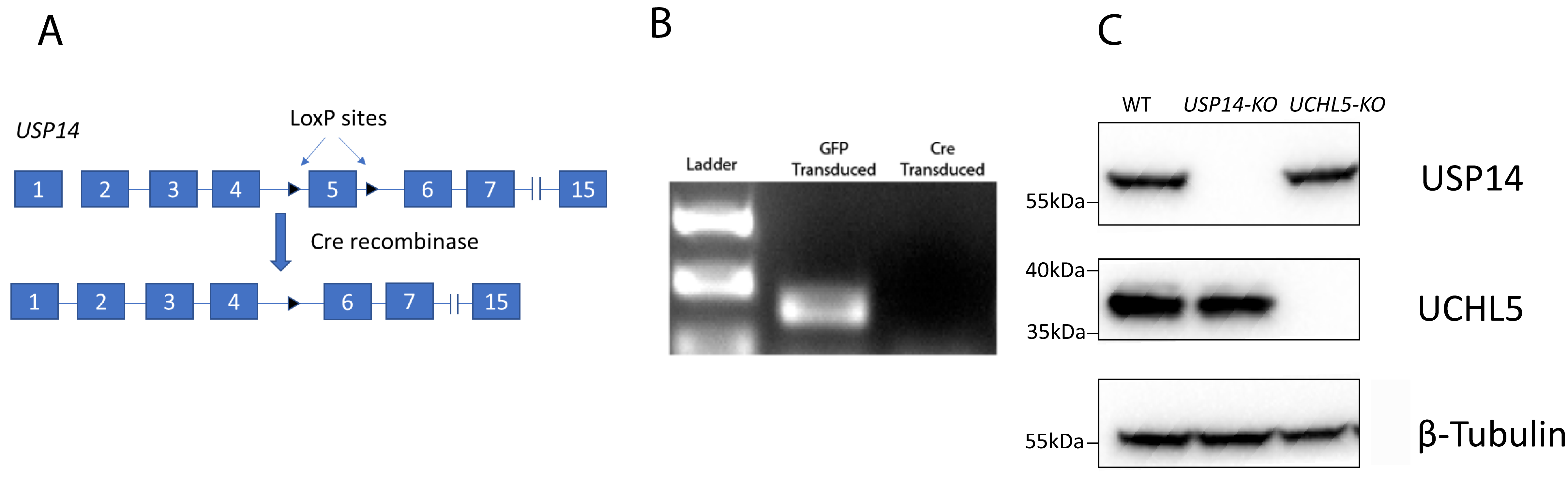

Supplement: Supplementary Figure 3 — Generation of DUB knockouts. (A) USP14 -/- cells were generated cells with floxed USP14. Transduction of cre recombinase results in the removal of the exon 5 and truncation of the USP14 protein. (B) Verification of USP14 exon 5 deletion by qPCR after cre transduction. (C) Presence of DUBs in the knockouts used. UCHL5 was knocked out through CRISPR/Cas9. [file Image_3.png]

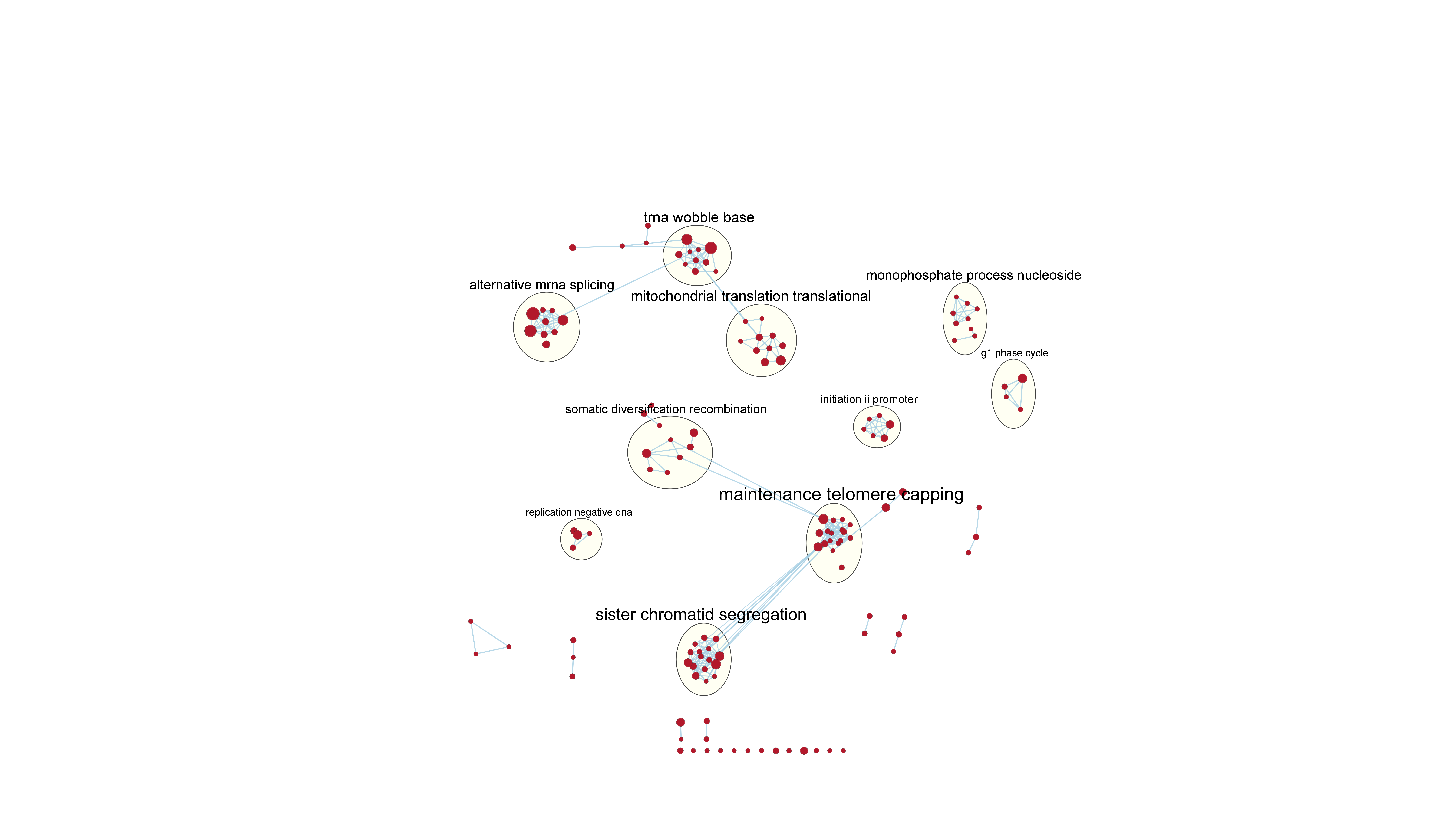

Supplement: Supplementary Figure 4 — Network analysis of enriched gene sets from the CRISPR dropout dataset. Individual gene sets are represented by solid circles. All gene sets presented here are overrepresented on guides that were enriched following bAP15 treatment. No gene set was significantly overrepresented on the depleted guides. The size of the circles correspond to the size of the gene sets. Gene sets connected with a blue line have overlapping genes. Width of the line represents degree of overlap between gene sets. [file Image_4.png]
